# Bluetooth Low Energy Mesh Networks: A Survey

**DOI:** 10.3390/s17071467

**Published:** 2017-06-22

**Authors:** Seyed Mahdi Darroudi, Carles Gomez

**Affiliations:** Department of Network Engineering, Universitat Polit̀ecnica de Catalunya, Castelldefels 08860, Spain; sm.darroudi@entel.upc.edu

**Keywords:** Bluetooth Low Energy, Bluetooth Smart, mesh networks, survey, Internet of Things, IoT, 6LoWPAN, 6Lo, Bluetooth Smart Mesh

## Abstract

Bluetooth Low Energy (BLE) has gained significant momentum. However, the original design of BLE focused on star topology networking, which limits network coverage range and precludes end-to-end path diversity. In contrast, other competing technologies overcome such constraints by supporting the mesh network topology. For these reasons, academia, industry, and standards development organizations have been designing solutions to enable BLE mesh networks. Nevertheless, the literature lacks a consolidated view on this emerging area. This paper comprehensively surveys state of the art BLE mesh networking. We first provide a taxonomy of BLE mesh network solutions. We then review the solutions, describing the variety of approaches that leverage existing BLE functionality to enable BLE mesh networks. We identify crucial aspects of BLE mesh network solutions and discuss their advantages and drawbacks. Finally, we highlight currently open issues.

## 1. Introduction

Bluetooth Low Energy (BLE), also marketed as Bluetooth Smart, has emerged as a major low-power wireless technology [1]. Leveraging a design that can reuse classic Bluetooth circuitry to a large extent, BLE has gained a dominant position in smartphones. This allows low-energy communication between the latter and other devices such as sensors, actuators, wearables, etc. [2]. Furthermore, the Internet Engineering Task Force (IETF) recently developed the adaptation layer to support Internet Protocol version 6 (IPv6) over BLE, thus facilitating the connectivity of BLE devices with the Internet of Things (IoT) [3].

While BLE is currently exhibiting high momentum, it is also facing significant challenges. A major drawback of a BLE network is limited coverage range since BLE was designed to follow the star network topology. For example, Wireless Home Automation Networks (WHANs) often require mesh topologies to enable communication between two end devices in a home. For this reason, technologies such as IEEE 802.15.4 (and thus Zigbee or Thread), Z-Wave, or Insteon, all of which support mesh networks, are being used in WHANs [4,5]. However, in such a relevant domain, BLE can only be used for point-to-point, single-link applications. A similar problem can be found in any scenario (e.g., industrial, urban, agricultural, etc.) where direct connectivity between any two endpoints may not be possible [6,7].

In order to cope with BLE network coverage limitations, two main approaches have been proposed by the community. The first one is based on reducing BLE Physical Layer signal bandwidth in order to increase link range while keeping the star topology network model, as in the recently published Bluetooth 5.0 specification [8]. However, this scheme still suffers from the hard coverage limitation of a star topology, i.e., extending the network coverage beyond one hop is not possible in such topology. Furthermore, a star topology network does not offer path diversity, which is a crucial property in wireless systems in order to cope with radio propagation impairments and node failures. The second approach relies on enabling a BLE mesh network. While this model involves the complexity of requiring mesh network mechanisms for end-to-end communication, it allows the coverage and path diversity limitations of a star topology to be overcome. These features have attracted the interest of academia, industry, and standards development organizations, which have developed or are developing numerous BLE mesh network solutions by following different techniques [9,10,11,12,13,14,15,16,17,18,19,20,21,22,23,24,25,26,27,28]. However, to the best of our knowledge, the literature lacks a consolidated view on this emerging area.

**Figure 1:**
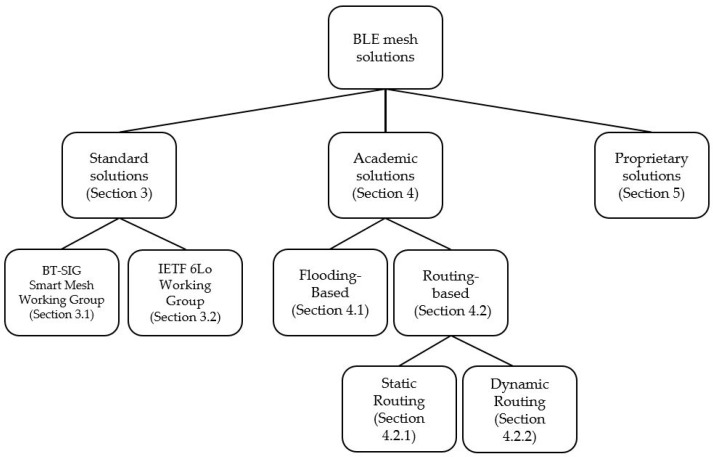
A taxonomy of Bluetooth Low Energy (BLE) mesh network solutions.

This paper comprehensively surveys BLE mesh network proposals. We provide a taxonomy for BLE mesh network solutions, whereby we have divided the identified solutions into three main categories, namely: standardized solutions, academic solutions, and proprietary solutions. The taxonomy, which is depicted in Figure 1, serves as the main structure for describing BLE mesh solutions in this paper. This structure provides the reader with homogeneous content in each main category, given the nature and the availability of information in each case. After reviewing the BLE mesh techniques considered, we critically discuss their main advantages and drawbacks and we present currently open issues.

In order to carry out the survey, we have considered and analyzed all existing BLE mesh network proposals and products to the best of our knowledge, as of the date of manuscript submission. In order to explore and find BLE mesh network solutions, we used web search tools (e.g., Google) as well as academic archives and digital libraries such as Institute of Electrical and Electronics Engineers (IEEE), Association for Computing Machinery (ACM), Multidisciplinary Digital Publishing Institute (MDPI), and Elsevier. We believe that, as a result, this survey is exhaustive.

The remainder of the paper is organized as follows. In Section 2, we introduce BLE, highlighting the features of the BLE specifications related to mesh topology support. Section 3, Section 4 and Section 5 give an overview of standard, academic, and proprietary BLE mesh network solutions, respectively. Section 6 discusses the main BLE mesh network approaches and presents open issues. Finally, Section 7 concludes the paper. 

## 2. Bluetooth Low Energy (BLE): Overview and Mesh Network Support

BLE was defined for the first time in 2010 by the Bluetooth Special Interest Group (SIG) as part of the Bluetooth 4.0 specification [2,29]. Since then, the following subsequent Bluetooth revisions have been published: Bluetooth 4.1, Bluetooth 4.2, and Bluetooth 5.0 [8,30,31] This section describes the main features of BLE for the aforementioned Bluetooth specifications, emphasizing the aspects related to mesh network topology support.

### 2.1. Bluetooth 4.0

**Figure 2:**
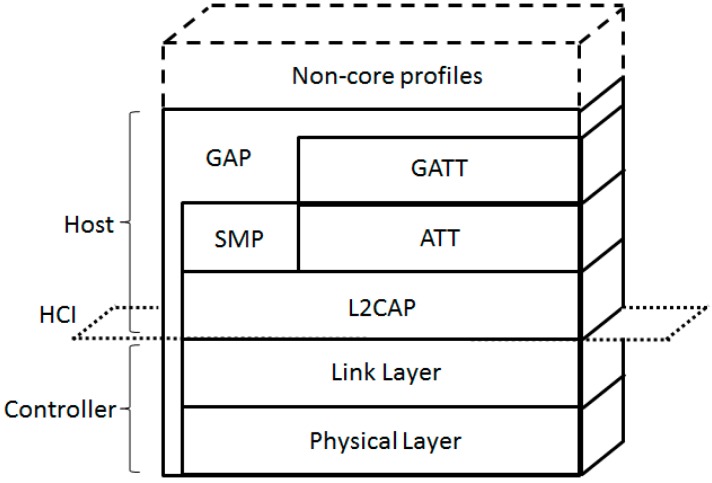
BLE protocol stack (adapted from [2]).

BLE defines a complete protocol architecture (Figure 2) with the purpose of enabling low power communication between devices. The architecture consists of two main parts; the Controller, which performs radio interface tasks, and the Host, which offers higher layer functionality and supports applications. The Controller consists of the Physical Layer and the Link Layer, while the Host comprises the Logical Link Control and Adaptation Protocol (L2CAP), the Attribute Protocol (ATT), the Generic Attribute Profile (GATT), the Security Manager Protocol (SMP), and the Generic Access Profile (GAP). The Host and the Controller communicate through the Host Controller Interface (HCI).

At the Physical Layer, BLE defines 40 Radio Frequency (RF) channels in the 2.4 GHz Industrial Scientific Medical (ISM) band. These channels are divided into three advertising channels, which are used for disseminating information, and 37 data channels, the purpose of which is the bidirectional exchange of messages between two devices. Adaptive frequency hopping is used over data channels. The data transmission rate is 1 Mbit/s.

BLE communication between two devices may be performed by following one of the two interaction patterns provided by the Link Layer. First, the two devices can act as an advertiser and a scanner, where the advertiser transmits data unidirectionally and the scanner can receive the data. In the second pattern, the advertiser and the scanner establish a bidirectional connection and adopt the slave and master roles, respectively. The master can have multiple connections in parallel with multiple slaves and is responsible for coordinating them through a Time Division Multiple Access (TDMA) scheme. Communication between a master and a slave takes place in connection events. The time between the start of two consecutive connection events for a given slave is equal to the value of a parameter called *connInterval*.

On top of the Link Layer, L2CAP provides connection-oriented and connectionless data services to the upper layer protocols with protocol multiplexing and segmentation and reassembly capabilities. On the other hand, application-layer interaction is handled by ATT and GATT. ATT defines server and client roles, where an ATT server exposes a set of attributes to an ATT client in a peer device. GATT defines a framework to use ATT in order to discover services and the exchange of characteristics from one device to another. A characteristic consists of a data set that includes values and properties. Furthermore, SMP provides key distribution to support secure identity and data encryption functionalities. Finally, GAP defines the generic procedures related to discovery, link management, and security aspects for communication between BLE devices.

Bluetooth 4.0 explicitly prohibits a slave node to participate in multiple connections simultaneously with other masters. Thus, the only network topology allowed for a BLE network based on the Bluetooth 4.0 specification is the star topology.

### 2.2. Bluetooth 4.1

The Bluetooth 4.1 specification was released in 2013. Bluetooth 4.1 incorporates a fundamental change with regard to BLE mesh network support; a device, regardless of its Link Layer role, can run multiple Link Layer instances simultaneously without limitation. Thus, a slave is allowed to be simultaneously connected to more than one master. In addition, one device can act as a slave at certain intervals and as a master at others, keeping parallel communications with its neighbors. This opens the door to creating extended network topologies beyond the star topology such as the mesh topology. However, the architecture and mechanisms for the formation and operation of a BLE mesh network are not defined in the Bluetooth 4.1 specification.

### 2.3. Bluetooth 4.2

Bluetooth 4.2, which was published in 2014, incorporates improvements mainly in three areas; Internet connectivity, improved security, and higher throughput. These updates are intended to increase the possibilities of BLE as a technology for the IoT. However, Bluetooth 4.2 does not provide further functionality to support BLE mesh networks.

### 2.4. Bluetooth 5.0

Published in late 2016, Bluetooth 5.0 has been the last Bluetooth specification released as of the writing of this piece. This new Bluetooth specification offers improvements in terms of range, data rate, and advertising channel functionality [32]. The latter comprises an advertising message capacity increase, along with the definition of two types of advertising channels, primary and secondary. Primary advertising channels are the same three advertising channels available in older BLE versions, while secondary advertising channels use the remaining 37 BLE channels (formerly defined solely as data channels). Note that secondary advertising channels can exploit frequency hopping like data channels do in any BLE version. However, like Bluetooth 4.2, it does not provide further functionality to support BLE mesh networks beyond those in Bluetooth 4.1 [8].

## 3. BLE Mesh Networks: Standardization

Industry interest in BLE mesh networks has triggered two standardization initiatives intended to enable communication between devices in BLE mesh networks. The two standards development organizations responsible for these efforts are the Bluetooth Special Interest Group (SIG) and the Internet Engineering Task Force (IETF), respectively. This section presents the status of both initiatives as of the writing of this article.

### 3.1. Bluetooth SIG: Bluetooth Smart Mesh

In early 2015, the Bluetooth SIG issued a press release announcing the creation of the Bluetooth Smart Mesh Working Group, the purpose of which is to develop an architecture to enable support for the mesh topology with BLE [33]. This initiative has enjoyed significant support since its inception, with 80 participating companies from a wide range of industries, including automotive, mobile telephony, industrial automation, home automation, and consumer electronics. Bluetooth SIG announced in their 2016 road map that mesh support was part of several enhancements to BLE to better support the IoT [34]. While the aim of the Bluetooth Smart Mesh Working Group is to build a common platform that can be useful for numerous use cases, home automation is probably the most clearly identified target application domain for BLE mesh networks as per current commercially available proprietary products (see Section 5). Bluetooth Smart Mesh can leverage the ubiquity of BLE in smartphones and other consumer electronic devices in the home.

The draft specification being developed by the Bluetooth Smart Mesh Working Group is not yet publicly available. However, Cypress and Mindtree demonstrated a mesh solution that met the latest version of the proposal under development by the Bluetooth Smart Mesh Working Group available in early 2016 and in early 2017, respectively [24,35] (see Section 5.6 and Section 5.10).

### 3.2. IETF: IPv6 over BLE Mesh Networks

In order to expand the IoT capillarity and its range of supported technologies, the IETF published the ‘IPv6 over Bluetooth Low Energy’ specification as RFC 7668 in late 2015 [3]. That specification adapted 6LoWPAN [36] in order to support IPv6 over BLE networks, although considering only the star topology [37].

With the aim to extend the functionality of RFC 7668 to enable IPv6 over BLE mesh networks, a new draft specification is being developed by the IETF IPv6 over Networks of Resource-constrained nodes (6Lo) working group [38]. This draft specification assumes that there exist Link-Layer connections between a node and its neighbors, through which IPv6 packets can be exchanged. Such connections are established by means of the Internet Protocol Support Profile (IPSP) [39]. As in RFC 7668, a 6LoWPAN-based adaptation layer is set below IPv6 and atop L2CAP. Such an adaptation layer provides IPv6 and User Datagram Protocol (UDP) header compression, which improves communication efficiency, and optimized IPv6 neighbor discovery, which offers network configuration suitable for constrained devices, both adapted for BLE mesh topologies. A routing protocol is assumed to find paths for communication between end devices. Routing is performed at the IP layer, although the routing protocol to be used is not determined by this draft specification.

## 4. BLE Mesh Networks: Academic Solutions

In the last few years, there have been various proposals from academia to enable BLE mesh networks. As of today, the number of proposals has grown steadily over time. We have classified them into two main categories; namely, flooding-based and routing-based solutions. The former do not perform routing, instead they broadcast packets throughout the network over BLE advertising channels. The latter use a routing protocol for packet forwarding and transmit data over BLE data channels. Table 1 summarizes the main characteristics of academic solutions for BLE mesh networks described in this paper, while Table 2 provides the main performance evaluation results reported by the authors of each solution (when available).

This section is divided in two parts. The first one overviews flooding-based solutions, while the second one focuses on routing-based solutions for BLE mesh networking.

### 4.1. Flooding-Based Solutions

We next examine two different academic solutions that are based on flooding to allow end-to-end data transmission in a BLE mesh network [9,10]. Note that several proprietary solutions also use flooding as the fundamental principle (see Section 5.1, Section 5.5, Section 5.7, Section 5.8, and Section 5.10).

A first flooding-based BLE mesh network proposal is provided in [9]. The authors studied how Packet Delivery Ratio (PDR) and latency can be bounded, while keeping energy consumption low. To this end, the authors devised a mechanism over Bluetooth 4.0 based on the Trickle algorithm [40]. Their approach follows gossiping [41], whereby traffic is propagated based on a given probability. This probability is determined by a node as a function that decreases with the node’s number of neighbors. In addition, the Trickle algorithm operates based on its own parameters to further filter the decision to rebroadcast traffic. The authors measured the current consumption of BLE nodes, as well as end-to-end packet latency.

On the other hand, a bounded flooding mechanism for BLE mesh networking, called BLEmesh, is presented in [10]. Bounded flooding limits rebroadcasting in intermediate nodes by only allowing a subset of these to participate in broadcasting operations. In BLEmesh, packets carrying data from a specific sender-destination couple are aggregated in batches. Data, together with control fields, which are used to decide which nodes will participate as broadcasters, are carried in the payload of advertisements. The control fields include two lists, namely, the Forwarder List and the Batch Map. The Forwarder List is a prioritized set of intermediate nodes in the path towards the destination, which is determined by the sender. The Batch Map identifies the last nodes that have broadcasted data corresponding to a specific batch. When a sender has data ready for transmission, the sender broadcasts the corresponding packet. Each intermediate node that receives a packet compares its priority in the Forwarder List with one of the last broadcasters shown in the Batch Map. If the node’s priority is higher than the one of the last broadcaster, the node sets its own address in the Batch Map as the last broadcaster and then rebroadcasts the packet (otherwise, the packet is dropped). BLEmesh uses the Expected Transmission Count (ETX) metric to set the Forwarding List value. The ETX assigns a cost to a link by estimating the number of transmission attempts needed for the successful delivery of a packet via that link [42]. Note that the Batch Map consumes space from already short-sized BLE packets. The authors compared their protocol with basic flooding and unicast source routing over Bluetooth 4.2. They show that BLEmesh requires fewer transmissions than basic flooding and unicast source routing, as considered in their work.

### 4.2. Routing-Based Solutions

This section gives an overview of academic routing-based solutions for BLE mesh networks. The section is divided in two parts, which focus on solutions that use static and dynamic routing, respectively. Note that several proprietary BLE mesh solutions are based on routing as well (see Section 5.2, Section 5.4, and Section 5.7).

#### 4.2.1. Static Routing Solutions

The authors in [11] present a solution that creates a static tree topology over Bluetooth 4.0. This scheme includes three kinds of nodes: (i) the root node, which is a central device as defined in the BLE specification; (ii) intermediary nodes, which actually comprise two subnodes (one acting as a master for nodes located in a lower hierarchical level and the other acting as a slave for nodes of higher hierarchical level); (iii) and leaf nodes, which are set to be peripheral devices. The authors define a simple hierarchy addressing scheme with two-byte addresses. The addressing scheme allows five tree levels, requiring a bigger address space for deeper networks. The transmission of data from nodes to the root takes place by sending the data from one node to the next one at a higher hierarchical level, and the process is repeated until data reach the root. The root can also send data to other nodes; in that case, the path is determined based on the destination and intermediate node addresses. Note that addresses in this solution are designed to reflect the hierarchical level and location of a node within the network. This solution is suitable for data collection in Wireless Sensor Network (WSN)s where the root node is a sink node. However, being a tree-based solution, this scheme suffers from the single-node failure problem and lacks a mechanism to rebuild the network after a node or link failure. The authors measured the power consumption, latency, and range of this solution experimentally.

Real Time BLE (RT-BLE) is another static routing solution designed over Bluetooth 4.1 and intended to enable bounded message delay for BLE mesh networks [12]. In RT-BLE, each node keeps a default route and an alternative route as a back up. RT-BLE connects subnetworks (comprised of a master and its slaves) in order to create an extended BLE network. However, there exist two limitations to network growth: (i) a node can establish a Link Layer connection with up to two masters and (ii) a master can establish a connection with at most another master, and, in this connection, the former shall play the slave role. In order to avoid an overlap of connection events for intermediate slave nodes that are connected to different masters, the authors used the Client Characteristic Configuration Descriptor (CCCD), a descriptor available in the GATT layer. CCCD acts as a switch and only allows one connection to be in active mode at a time, while the rest are kept inactive. The authors analytically calculate the latency in a subnetwork and the end-to-end latency between devices in different subnetworks. They also provided experimental inter-subnetwork results by using devices with the X-NUCLEO-IDB05A1 chip from STMicroelectronics.

#### 4.2.2. Dynamic Routing Solutions

Five different dynamic routing-based solutions for BLE mesh networks are presented in this subsection. The solutions vary in the routing approach and in the use of advertising channels, data channels, or both for finding routes.

##### MHTS

The first BLE mesh solution, called MultiHop Transfer Service (MHTS), was published in 2013 [13]. MHTS was designed over Bluetooth 4.0, based on next-hop, on-demand routing over the GATT layer. MHTS consists of two phases. The first one handles neighbor discovery, connection establishment, and route discovery. The second one comprises data storing and forwarding over the end-to-end path.

During the first phase, neighbor discovery and connection establishment are performed by using common BLE mechanisms. Route discovery is carried out when a packet is ready to be sent but a route to the intended destination is not available in the sender routing table. To initiate route discovery, the sender transmits an advertisement, which carries the target destination, the maximum number of hops between sender and destination (as set by the sender), and the maximum time for the route discovery process. If a neighbor that receives the advertising packet does not know a route to the destination, the neighbor proceeds like the sender and transmits advertising packets requesting for a route towards the destination. This process is repeated until a node that knows a route to the destination is found, which then establishes a Link Layer connection with its precursor neighbor. The latter creates a routing table entry that indicates the neighbor as the next hop towards the destination, the neighbor performs the same operation with its own precursor neighbor, and the procedure is repeated until the source node updates its routing table with the discovered route.

In the second phase, every node in an end-to-end path transmits data as a slave in the Link Layer connection with its next hop, while the latter plays the role of a master in that connection. Since in Bluetooth 4.0 only a star topology is allowed, the above scheme is achieved by transferring the whole data unit (e.g., a large file) in a per-hop basis, as follows. In the first link of the end-to-end path, a connection is established between the sender and the receiver; then the whole data unit is transferred from the sender to the receiver, and the connection is then released. The same procedure is carried out in subsequent links, until the (possibly large) data unit reaches the destination. Note that, in this solution, a node never participates simultaneously in two connections. However, end-to-end transmission is limited by the available memory of BLE devices. With the resources of the CC2640 chip, MHTS can transmit packets over up to five hops for a file size of 1 kB [43]. For networks of greater diameter, devices need a larger amount of memory.

##### BMN

BLE Mesh Network (BMN) is another routing-based solution that transmits routing messages via advertising channels. BMN uses the Directed Acyclic Graph (DAG) structure as a basis for routing [14,15], inspired by the IPv6 Routing Protocol for Low power and lossy networks (RPL) [44]. BMN was designed over Bluetooth 4.1, and its operation consists of three phases; namely, construction, maintenance, and optimization.

The construction phase has the goal of establishing Link Layer connections between neighboring devices, determining nodes’ parents, and creating routing tables. A parent is the next hop for a node in its path towards the DAG root, and thus the parent of a node is placed in a higher hierarchical location than the node. When a node wants to join a network, it transmits DAG Information Solicitation (DIS) messages to announce its presence and solicit DAG information. DAG Information Object (DIO) messages are sent by neighboring nodes in response to DIS messages. Based on the DIO messages received, the node must determine a parent and an alternative parent. A parameter named Rank is defined to specify the quality of routes between nodes and the DAG root. Nodes with lower Rank values are parent (and alternative parent) candidates for new nodes. In BMN, Rank is computed based on nodes’ residual energy and distance towards the DAG root. Each node maintains a table where it stores its parent, its alternative parent, and the list of its children (i.e., the nodes that have chosen this one as their parent). On the other hand, the root has a routing table that stores routes for all possible destinations in the network.

The maintenance phase aims to improve BMN parameter settings and forward packets to their intended destinations. To forward a packet, each source node first looks for a route to the destination in its routing table. If a route is not found, the node sends the packet to its parent. The parent performs the same process, which is repeated until a route to the destination is found. In the worst case, a packet must be sent to the DAG root to be routed to its destination. The optimization phase has the purpose of node weight balancing so that all nodes in the network have nearly equal distance to the DAG root.

BMN sends data messages over data channels, while control messages are sent through advertising channels. Being based on a DAG structure, this solution may suffer issues similar to those of tree-like networks such as single-node failure (although it is mitigated by alternative parents when available) and congestion in the area close to the root. The authors measured the power consumption, latency, and data loss experimentally over a network composed of smartphones [14,15].

##### On-demand Scatternet Formation and Routing

In [16], the authors present a protocol for forming scatternets and on-demand routing for Bluetooth 4.1. Scatternets are network topologies composed of interconnected piconets, while the latter are simple star topology networks comprised of a master and its slaves. In order to interconnect piconets, the nodes may act as both a master and a slave. This protocol consists of two phases; scatternet formation and route discovery.

In the scatternet formation phase, masters create a list of their connected slaves and vice versa. Nodes acting as both roles elaborate both slave and master lists. In order to allow connection establishment between a new node and its neighbors, the node alternates scanning and advertising states. The node assumes the master or the slave role, depending on its role as a scanner or an advertiser, at connection establishment time.

In route discovery, the source node first sends a route request to its master. If the target destination is not in the slave list of the master, the latter initiates a breadth-first search by forwarding the route request to any slave in its piconet that participates in another piconet. Such slaves resend the route request to their masters in other piconets. This process will continue until the destination is found. By following this process, different routes to a destination are obtained. After collecting all possible routes to a destination, the source node exploits the shortest one and saves it in a route cache. However, network resources are wasted since only one of the discovered routes is used. Another challenge of this mechanism is scalability; in route discovery, the breadth-first search continues until the destination is reached, while in other solutions (e.g., [13]), route discovery can be completed once an intermediate node with a route towards the destination is found.

The authors evaluated message delay and network throughput experimentally.

##### Mediation Service over Named Data Networking

The authors in [17] use Named Data Networking (NDN) [45] to support BLE mesh networks. The Bluetooth version assumed is 4.1. The NDN paradigm changes the networking focus from identifying locations (e.g., as in IP networks, whereby an endpoint communicates with a specific destination where data of interest are) to identifying the content itself for data retrieval, regardless of its location. NDN names every chunk of data with an appropriate Uniform Resource Identifier (URI), and operates over a distributed database, which allows how an endpoint can retrieve data of interest to be determinded.

The authors leverage GATT services, characteristics, and attributes as the database over which they apply NDN for BLE mesh networks. A Mediation Service [46] is utilized in order to aggregate distributed databases. In this solution, each slice of data is uniquely identified by Universally Unique Identifiers (UUIDs). Two kinds of packets are transferred through the BLE mesh network; Interest and Data. A device requesting data (e.g., the reading of a temperature sensor) sends an Interest packet, and the result is returned in a Data packet following the same route backwards. In order to avoid Interest loops, authors use a Nonce Descriptor, which is a 32-bit random number assigned to each Interest packet. The concept has been proofed by the authors over different hardware platforms, but the solution has not actually been evaluated.

##### ALBER

Similarly to BMN, another solution based on a DAG structure (in this case created by the RPL protocol), called Adaptation Layer between BLE and RPL (ALBER), has been proposed over Bluetooth 4.1 [18]. ALBER performs four different tasks: (a) broadcasting RPL control messages through BLE advertising channels; (b) broadcasting RPL routing metrics values that reflect BLE link qualities; (c) transmitting routing table updates; and (d) managing parent changes in order to prevent packet loss.

In order to determine the Rank of a node, in this solution nodes use a metric inspired by ETX. Since the Link Layer in BLE does not provide retransmission information to upper layers, ALBER requires the L2CAP layer to transmit ping packets to parent nodes and uses the obtained Round-Trip Time (RTT) measurements to calculate the Rank value for this node. The authors found experimentally that RTT in BLE can be computed in terms of *connInterval* periods. Thus, the authors defined a new metric called Expected number of Connection Intervals (ECI), which reflects RTT expressed in terms of *connInterval* periods.

The authors performed an experimental evaluation of their solution, focusing on three main objectives: (i) comparing RPL over BLE with RPL over IEEE 802.15.4; (ii) determining the impact of the *connInterval* setting; and (iii) evaluating the effect of ECI on the performance of RPL over BLE. Regarding the first objective, the authors obtained better PDR with RPL over BLE than over IEEE 802.15.4, which they attributed to the adaptive frequency hopping mechanism used in BLE. Moreover, spasmodic PDR degradation in IEEE 802.15.4 caused frequent parent changes. In relation to the second objective, the authors found that PDR performance was improved by reducing *connInterval*. However, increasing *connInterval* decreases energy consumption. Finally, the authors found that using ECI reduces the amount of parent changes and improves PDR.

## 5. BLE Mesh Networks: Proprietary Solutions

In order to exploit market opportunities for BLE mesh network products, several companies have developed proprietary solutions for BLE mesh networks. A majority of these solutions have been designed for the fields of home automation and/or lighting. However, they may also be suitable for other use cases. We next present a comprehensive set of commercial, proprietary BLE mesh network solutions. Due to the proprietary nature of these solutions, the availability of details on the mechanisms used by these solutions is limited. Their main features are summarized in Table 3.

### 5.1. CSRmesh

Cambridge Silicon Radio (CSR) developed CSRmesh, a proprietary protocol that operates on top of Bluetooth 4.0 and subsequent, which allows messages to be forwarded across BLE devices in a mesh topology [19].

CSRmesh uses flooding over advertising channels for end-to-end communication. A flat model is used, whereby all devices have the same hierarchical level. Flooding is controlled by using a Time To Live (TTL) mechanism and by preventing rebroadcast of the same packet more than once [47]. A CSRmesh network can in theory comprise up to 64,000 devices. Messages can have individual or group recipients. CSR offers CSR101× modules, which support CSRmesh and the CSRmesh Development Kit, which provides a set of assessment tools and software development for CSRmesh.

Among the proprietary BLE mesh network solutions, CSRmesh appears to be the most popular one, as witnessed by the amount of academic work that is based on this solution [47,48,49].

The authors in [47] evaluated a BLE mesh network composed of 10 CSRmesh nodes in a building. Performance metrics such as single-hop and multi-hop PDR were analyzed experimentally.

Another CSRmesh case study was presented in [48], although, unlike the prior, simulation results were provided in addition to experimental results. The authors evaluated empirically different network scenarios with 19 CSRmesh modules. The maximum PDR was found to be up to 90% for short distances between sender and receiver. The authors showed the benefits of mesh networking to increase PDR over relatively long distances. They also identified a PDR trade-off that depends on the sender packet rate, wherein PDR is maximized for a sender rate of four packet/s.

The same work included the simulation of a 500-node network, whereby nodes were spatially located following a uniform grid pattern and each node had nine direct neighbors. The authors showed that network size did not have a significant effect on overall end-to-end PDR for the considered node spatial distribution. However, since such node distribution is not usual in realistic mesh network deployments, the authors also evaluated a randomly distributed mesh network, in which single-node failure and bottleneck problems were found.

A further academic case study based on CSRmesh can be found in [49]. The authors first evaluate CSRmesh and then improve basic CSRmesh mechanisms by developing two different solutions; Individual Mesh and Collaborative Mesh. Nodes of the first type only transmit their own data and do not relay packets from other nodes, while nodes of the latter type transmit their own packets and also relay the packets received from other nodes. In addition, a new packet format was designed. These improved mechanisms are compared by the authors with the following two approaches; a Bluetooth 4.0, non-mesh solution and a mesh solution using basic CSRmesh functionality.

### 5.2. BLE-MESH.com

BLE-MESH.com is a start-up that recently created a solution that offers BLE multihop networks. This solution consists of mesh nodes and a mesh gateway, compatible with third party devices. Both mesh nodes and the gateway implement the BLE-MESH routing protocol [20].

### 5.3. Wirepas and Nordic Semiconductor

Nordic Semiconductor released in 2014 a rebroadcasting mesh solution, namely nRF OpenMesh, for nRF5× family modules [50]. Subsequently, in late 2014, Wirepas and Nordic Semiconductor presented a solution for mesh networks for the nRF51822 BLE chip called Wirepas Pino. This is a proprietary solution that can support a high density of nodes and through which the network topology is continuously self-optimized [21].

### 5.4. NXP

In 2015, NXP (Eindhoven, Netherlands) demonstrated a proprietary mesh solution for BLE modules. This solution is based on a synchronized mesh network and routing using BLE. In this solution, each node has its own routing table. Such functionality is available for the QN9020 platform [22].

### 5.5. Silvair

Silvair developed a proprietary solution for BLE mesh networking, which has been included in smart lighting products. Silvair is one of the major contributors to the development of the Bluetooth specification to the Bluetooth SIG Smart Mesh Working Group.

The Silvair solution adds functionality to GATT services and allows direct communication between peripherals (i.e., slaves in traditional BLE), allowing communication over up to 63 hops. One of the concepts used in this solution is connectionless communication [23].

### 5.6. Cypress

In early 2016, Cypress demonstrated a solution and an implementation for lighting applications that was compliant with the latest proposal at the time from the Bluetooth Smart Mesh Working Group. Cypress offered to be used in health and fitness equipment, home appliances, and toys [24].

### 5.7. Ilumi MeshTek

MeshTek is a very recent BLE mesh solution developed by ilumi solutions. In contrast with other proprietary solutions, it can work in two modes. First, it allows packets to be broadcast through advertising channels. Secondly, it enables large data packet transfer over a connection-oriented mechanism. Different MeshTek device models are available for different mesh use cases [25].

### 5.8. Estimote Beacons

Estimote introduced their BLE mesh solution with the aim to develop a platform for serving Bluetooth beacons. Since Bluetooth beacons use advertisements to transport data, this solution uses broadcasting to enable mesh communication. These devices, which announce the readings from sensors of various types, are interconnected by means of a mesh network topology. The latter allows control and management settings to be propagated throughout the whole network [26].

### 5.9. Telink Semiconductor

Telink Semiconductor presented their first BLE mesh lighting solution in early 2016 [51]. Their multi-standard wireless System-on-Chip (SoC) product (i.e., TLSR8269F512) offers BLE mesh networking over Bluetooth 4.2 [52]. This BLE mesh network solution supports concurrent management (e.g., a lighting system can be simultaneously configured and managed by different entities without conflict), group management (i.e., a group of devices can be managed), and real-time message delivery (for a network of up to 200 nodes [27]).

### 5.10. Mindtree Bluetooth Mesh

Mindtree has developed a solution for BLE mesh networks. The company claims its Bluetooth Mesh's IP to be aligned to Bluetooth SIG’s draft Bluetooth Smart Mesh specifications. According to the available information on this solution, it supports all mandatory and optional specifications, all states and topology roles, and flooding-based operation. Moreover, it offers application-level encryption. Mindtree products using BLE mesh solutions comprise BlueLitE and EtherMind Bluetooth software [28].

## 6. Discussion and Open Issues

In the last two sections, we have presented academic and proprietary BLE mesh network solutions. Based on this review, we next discuss fundamental aspects for the design and performance of BLE mesh networks. We then identify currently open issues.

### 6.1. Discussion 

Different models may be devised for a BLE mesh network solution. The advantages and drawbacks of different BLE mesh network solution properties are described in this subsection. In particular, we discuss the use of a flooding or a routing paradigm, routing approaches, the relationship between the types of channels used (i.e., advertising or data channels), reliability, large data unit support, and suitability for IPv6 support.

#### 6.1.1. Flooding vs. Routing

There exist two multihop paradigm fundamental categories in BLE mesh networks, flooding and routing. An advantage of flooding is its simplicity, as neither it requires the establishment of connections between neighboring devices nor a routing protocol. This avoids delays due to route creation, as well as the complexity and memory consumption due to routing tables and their maintenance. However, since data are flooded throughout the network, this approach may be inefficient in terms of the total number of messages sent by network nodes for the purpose of end-to-end communication between two devices. Such inefficiency increases with network size, thus longer sized networks will benefit from routing. Nevertheless, mitigation techniques (such as Trickle [9] or node-density-aware rebroadcasting [10]) allow the message overhead of flooding-based solutions to be limited.

Another aspect to be considered is the data message rate that needs to be supported. If data transmissions are infrequent, flooding overhead might compensate the cost of creating and/or maintaining a routing structure.

#### 6.1.2. Routing Approach 

Two main types of routing solutions are used by routing-based BLE mesh network solutions: (i) tree/DAG proactive-routing [11,12,14,15,18] and (ii) on-demand routing [13,16]. Solutions of the first category are suitable for sensor data collection applications, where there exists one main destination (i.e., the tree/DAG root) that may be a sink node and/or a gateway to other networks. Such structure is also suitable for communication from a central device (e.g., a remote control) to the rest of the devices in the network. However, it is not optimized for communication between any pair of devices in the network. On-demand routing is not limited to any particular underlying structure, and thus it can find optimal routes between any two nodes. However, with this model, routing table size increases with the number of destinations for which data are sent. In this routing category, routes are searched only when data have to be sent. Remarkably, proactive routing is only used as part of tree/DAG-routing solutions, since otherwise the routing state will increase with the total number of nodes in the network.

#### 6.1.3. Reliability: Advertising Channels vs. Data Channels

BLE mesh network solutions make use of advertising channels and/or data channels in different ways. The choice of each channel type has an important impact on performance in terms of data transmission reliability.

Flooding-based solutions (which use advertising channels) [9,10,19,20,23,26,28], being based on Bluetooth 4.x, will not be able to exploit frequency hopping over the 37 BLE data channels. Therefore, such solutions will not benefit from a good mechanism for robustness. This is particularly critical in smart home environments, where interference from devices using other technologies and multipath propagation is a common issues [4]. However, the use of data channels (and their frequency hopping mechanism) requires the establishment of Link Layer connections between neighboring devices. Another approach, only available in Bluetooth 5.0, is using secondary advertising channels, which allows frequency hopping to be exploited for such channels.

On the other hand, routing-based solutions that use advertising channels to send routing protocol messages but use data channels for data transmission [13,14,15,18], may suffer the issue of finding routes based on channels for which conditions (in terms of link qualities) may be different from the ones that will be encountered by data transmission.

Finally, the use of data channels allows Link Layer reliability to be exploited, which provides Link Layer acknowledgments and retries. In contrast, advertising channels are limited to broadcasting, which does not use any mechanism for enhanced reliability.

#### 6.1.4. Large Data Unit Support

In order to transmit large data units (i.e., larger than the maximum Link Layer payload size) over a BLE mesh network, solutions that use data channels for data transmission can leverage functionality such as Segmentation and Reassembly (SAR), which is provided by L2CAP [11,12,13,14,15,16,17,18]. However, the transmission of large data units cannot be done over advertising channels with currently existing BLE functionality. This fact excludes flooding-based solutions, which only use advertising channels, for use cases in which large data units (e.g., large packets or large files) need to be sent over BLE mesh networks. Note that given the success of BLE beacons, Bluetooth 5.0 has increased the advertisement payload size supported up to 255 bytes, compared with previous Bluetooth versions. This feature mitigates the problem described, yet flooding-based solutions do not support the transmission of data units larger than 255 bytes.

#### 6.1.5. Suitability for IPv6 Support 

RFC 7668 defines an adaptation layer below IPv6 and over L2CAP. This design assumes that IPv6 packets will be sent over Link Layer connections, which use data channels. Therefore, BLE mesh network solutions that use advertising channels for data transmission are not compatible with RFC 7668 or its extension for BLE mesh networks. In addition, since no routing solution for use below IP has been standardized by the Bluetooth SIG, the current IETF draft specification for IPv6 over BLE mesh networks requires IP routing. Therefore, if a routing protocol is used, routing protocol messages need to be sent over L2CAP and Link Layer connections. Only BLE mesh solutions that use data channels for sending control (e.g., routing protocol) messages or solutions that rely on static routing are compatible with this requirement [11,12,16,17] .

### 6.2. Open Issues

Currently, there exist several open issues in areas that have not yet been deeply considered for BLE mesh network solutions. These include security, the effects of address assignment on privacy and routing performance, multicast, and interoperability. 

#### 6.2.1. Security

Security is of the utmost importance in IoT networks, given the impact that compromising such networks may have on physical world activities. However, security is currently a challenge in BLE mesh networks.

Since SMP services are not available over advertising packets, only packets transmitted through data channels are encrypted. Moreover, authentication is only performed within a connection. Thus, routing and data packets that are transmitted through advertising channels are not secured unless the application layer provides a security solution.

On the other hand, since BLE was originally designed for star topology networks, data channels are protected by per-hop security. End-to-end encryption and authentication are not currently supported in BLE mesh networks. 

Future IPv6-based BLE mesh solutions will be able to leverage existing end-to-end security functionality for IP-based protocols. A first option is Transport Layer Security (TLS), which is commonly used to secure HTTP communications [53]. Furthermore, specific work has been recently done on Datagram TLS (DTLS) for constrained environments [54], as in fact DTLS is the default security protocol for the Constrained Application Protocol (CoAP), a lightweight application-layer protocol that has been designed for constrained devices and is therefore a good candidate for BLE devices [37]. Object security is another recent approach being developed for securing application-layer payloads (i.e., end-to-end) [55], which may be suitable for BLE mesh networks as well. Non-IP-based BLE mesh networks will need to develop end-to-end security functionality that might be equivalent to the approaches described. 

#### 6.2.2. Privacy vs. Routing

BLE devices can use privacy addresses, which are updated frequently, in order to counter threats such as activities correlation over time, location tracking, or exploiting vendor-specific vulnerabilities [56]. However, this approach may have negative impact on a routing mechanism. Since BLE private addresses are often changed, routing tables should be updated frequently as well, potentially leading to increased routing protocol message overhead. There will be additional routing protocol message overhead as long as the frequency of address updates exceeds the intended rate of routing protocol messages. A possible future approach to avoid this issue might be a coordinated, network-wide scheme whereby nodes share information that allows the address in use for each node at a given moment to be determined. Such a mechanism has not been developed as of the writing of this article to the best of our knowledge.

#### 6.2.3. Multicast

Multicast is an important paradigm for use in BLE mesh networks. Some applications require multicast such as turning on a specific group of lights in a building or polling a specific group of sensors. Efficient communication with a group of destinations is crucial in this type of environment.

However, since the BLE Link Layer does not support multicast, transmitting data to a subset of neighbors in a BLE mesh network translates into unicasting data as many times as needed. If the group of multicast destinations is large compared with the network size, flooding-based solutions could be suitable. Nevertheless, the efficiency of this scheme will decrease as the number of multicast group members deviates from the network size. 

Another approach for multicast in BLE mesh networks is leveraging layer three multicast, e.g., IPv6 multicast in IPv6-based BLE mesh networks, jointly with a filtering tool. The latter may benefit from a table in each node containing information on which specific neighbors of the node need to receive a multicast packet (for further forwarding or as actual recipients of the packet). This solution would reduce inefficiency due to the lack of BLE Link Layer multicast, although it will not be possible to avoid the specific issue of sending a multicast packet from a node to some or all of its neighbors by unicasting it to as many of these neighbors as needed. The overhead for maintaining the multicast infrastructure should also be considered. In this regard, an interesting proposal called constrained-cast has been recently adopted by the IETF Routing Over Low power and Lossy networks (ROLL) working group in order to enable efficient multicast in RPL networks, based on use of bloom filters [57].

#### 6.2.4. Interoperability

The reviewed academic and proprietary solutions are not conceived to be interoperable. In the first case, the intent is to demonstrate the feasibility of a paradigm for research purposes. In the second one, the purpose is to provide a commercial product, regardless of compatibility with the developments offered by other companies. However, interoperability is crucial to allow products of different manufacturers to communicate with each other, and standardization is required to enable this paradigm. The completion of the two standardization items in progress described in Section 3 will be fundamental to achieve interoperability for BLE mesh networks. However, the interoperability of existing BLE mesh network solutions is an open issue as of the writing.

Among the two standardization items, the IETF one is based on using IP, while no evidence has suggested so far that Bluetooth SIG’s Bluetooth Smart Mesh will also be. Nowadays, constrained devices are being connected to the Internet by means of two main approaches; (i) IP-based and (ii) non-IP-based (via protocol translation gateways). While the latter was the main approach more than a decade ago, in the last years the market has changed towards IP convergence. It is reasonable to consider that the market presence of IP-based technology for constrained devices will continue to increase, since this facilitates interoperability, scalability, and application development. 

As stated in 6.1.5, IPv6-based BLE mesh solutions require the use of data channels for the transmission of both routing and data messages. Among these, static solutions do not appear to be solid candidates given the intrinsic dynamics of mesh networking. Therefore, solutions based on dynamic routing that use data channels for the transmission of both routing and data messages, e.g., [16,17], are currently the most promising ones to unleash the potential of BLE mesh networks.

## 7. Conclusions

BLE mesh networking is an emerging area with the potential to expand the BLE applicability space. In the last few years, BLE mesh network academic solutions have been proposed, and proprietary solutions have been released. As of the writing of this article, standards for BLE mesh networking are being developed.

BLE mesh networking solutions are diverse. Flooding-based schemes favor simplicity, while routing-based solutions are more elaborate. These two solution categories use advertising channels and data channels, respectively, for data transmission. While using data channels requires Link Layer connection establishment between neighboring devices, this approach allows BLE Physical Layer features (e.g., adaptive frequency hopping), Link Layer features (bidirectional communication and reliability), and L2CAP layer features (segmentation and reassembly) to be exploited, all of which are not available for advertising-channel-oriented data transmission.

There exist several open issues in BLE mesh networking. We suggest that the community should focus on solving problems in security, multicast, and interoperability in order to deliver secure and high quality BLE mesh networks.

## Figures and Tables

**Table 1:** Main features of academic BLE mesh solutions. Note that for a given category, solutions are ordered based on the Bluetooth version used (first) and on the year (secondly).

		Proposal Reference	Proposal Name	Year	Multi-Hop Paradigm	Bluetooth Version	Type of Channels	
							Advertising Channels	Data Channels
**Flooding**		[9]	N/A	2016	Trickle + gossiping	4.0	Data	-
		[10]	BLEmesh	2015	Bounded flooding	4.2	Data	-
**Routing**	**Static**	[11]	N/A	2014	Tree-based routing	4.0	-	Data
		[12]	RT-BLE	2016	Pre-configured	4.1	-	Data
	**Dynamic**	[13]	MHTS	2013	On-demand routing	4.0	Routing	Routing/Data
		[14,15]	BMN	2015	DAG-based routing	4.1	Routing	Data
		[16]	N/A	2015	On-demand routing	4.1	-	Routing/Data
		[17]	N/A	2015	Named Data Networking	4.1	-	Routing/Data
		[18]	ALBER	2016	DAG-based routing	4.1	Routing	Data

**Table 2:** Performance reported for BLE mesh network academic solutions.

Proposal	Multi-Hop Paradigm	Evaluation Platform	Performance	
			Metrics	Results/Conclusion
[9]	Trickle + gossiping	nRF51822 SoC based on ARM Cortex M0	Latency	20 s (3 hops)
			Node lifetime	589 days (6000 mAh battery, 5% duty cycle)
[10]	Bounded flooding	TI CC2540	Packet overhead	16 packets (BLEmesh), 25 packets (source routing), 96 packets (flooding) Note: packet transmission over 5 hops
[11]	Tree-based routing	TI CC2540, SMARTF05 EB	Latency	1.0 s (1 hop), 1.3 s (2 hops), 2.1 s (3 hops)
			Node lifetime	202 days (peripheral), 60 days (central with 3 connected peripherals) Note: *connInterval* = 500 ms
[12]	Static routing	X-NUCLEO-IDB05A1 (STM)	Latency	<0.35 s (5 hops)
[13]	On-demand routing	TI CC2540	Latency	25 s (first packet), 0.79 s (rest of packets) Note: 22-byte packet over 3 hops
[14,15]	DAG-based routing	Smartphone	PDR	98.8% (512-byte file, 5-hop path)
			Latency	2.09 s (2 hops), 2.90 s (3 hops), 3.54 s (4 hops), 4.00 s (5 hops)
			Avg. current consumption	210.9 mA (sender), 228.7 mA (receiver), 234.6 mA (relay) Note: 10-100 kB file transmission
[16]	On-demand routing	Broadcom BCM434x (iPhone 6)	Throughput	~6 kbit/s (10-node network)
			Latency	<5 s (10-node network)
[17]	Named Data Networking	S130 Nordic, TI CC2540, custom prototype	N/A	N/A
[18]	DAG-based routing	MSP430 microcontroller, TI CC2420, Broadcom BCM4356	PDR	~100% (*connInterval* = 50 ms) ~80% (*connInterval* = 200 ms)
			Comparison with IEEE 802.15.4	BLE mesh network provides greater PDR, lower number of parent changes and lower overhead
			Impact of ECI metric	Greater PDR (~100%) and lower parent changes than without ECI

**Table 3:** Summary of BLE mesh network proprietary solutions and intended applications (as claimed by each corresponding manufacturer).

Proposal	Name	Year	Mesh Paradigm	Intended Application
[19]	CSRmesh	2014	Flooding	Lighting, HVAC, switch manager, physical access authorization, smart home
[20]	BLE-MESH.com	2014	Routing	Smart city and home automation.
[21]	Wirepas and Nordic Semiconductor	2014	N/A	Smart home, smart city, smart door lock, lighting, sport, fitness, health, virtual reality
[22]	NXP	2015	Routing	Smart home, smart city, lighting
[23]	Silvair	2016	Flooding	Smart lighting
[24]	Cypress	2016	N/A	Health, fitness, home appliances, toys
[25]	Ilumi MeshTek	2016	Flooding/Routing	Smart home, remote control, health monitor
[26]	Estimote	2016	Flooding	Beacons for motion detection, guiding.
[27]	Telink Semiconductor	2016	N/A	Lighting, home automation, smart office, smart cities, remote controls, human interface devices, wearable devices
[28]	Mindtree Bluetooth Mesh	2016	Flooding	Multi-purpose
